# Improvement of quality of life and psychological distress after inpatient cancer rehabilitation: Results of a longitudinal observational study

**DOI:** 10.1007/s00508-017-1266-z

**Published:** 2017-09-15

**Authors:** David Riedl, Johannes M. Giesinger, Lisa M. Wintner, Fanny L. Loth, Gerhard Rumpold, Richard Greil, Alain Nickels, Thomas Licht, Bernhard Holzner

**Affiliations:** 1 Department of Psychiatry, Psychotherapy and Psychosomatics, Medical University of Innsbruck, Innsbruck, Austria; 2 Innsbruck Institute of Patient-Centered Outcome Research (IIPCOR), Innsbruck, Austria; 3 University Clinic of Internal Medicine III, Paracelsus Medical University Salzburg, Salzburg, Austria; 4 Oncological Rehabilitation Center St. Veit, St. Veit im Pongau, Austria

**Keywords:** Rehabilitation, Cancer, Health-related quality of life (HRQOL), Psychological distress, Inpatient

## Abstract

**Background:**

With the growing number of cancer survivors worldwide the need for high quality cancer rehabilitation after primary treatment is steadily increasing. The aim of the present study was to investigate change of psychological distress and health-related quality of life (HRQOL) during multidisciplinary inpatient cancer rehabilitation in a large sample of cancer survivors suffering from different cancer entities.

**Methods:**

We analyzed data from routine HRQOL and distress monitoring at a cancer inpatient rehabilitation center. Cancer survivors completed the European Organization for Research and Treatment of Cancer (EORTC) Quality of Life Questionnaire Core-30 (EORTC QLQ-C30) and the Hospital Anxiety and Depression Scale (HADS) before and after multidisciplinary rehabilitation treatment. Changes of patients’ functioning and symptoms were analyzed using repeated measures analysis of variance (ANOVA) and effect sizes (Cohens’ d). Patients’ pretreatment and posttreatment scores were compared to reference data from the German general population.

**Results:**

A total of 939 patients (mean age 58.6 years, SD 11.9 years; 59.9% women) who attended rehabilitation from January 2014 to September 2015 were included in the analysis. We found clinically meaningful improvement in almost all domains of the EORTC QLQ-C30 as well as in anxiety and depression (HADS). The largest improvements were found for the QLQ-C30 subscales emotional functioning (d = 0.78), fatigue (d = 0.65), and social functioning (d = 0.56).

**Conclusions:**

We found clinically meaningful improvements of patients’ HRQOL, anxiety and depression during an oncological inpatient rehabilitation treatment. Our results warrant further prospective controlled studies to evaluate the long-term effectiveness of inpatient rehabilitation.

## Introduction

Survival rates of cancer patients have steadily improved over the last 20 years, thanks to better diagnostics and advances in treatment. This leads to new challenges in cancer treatment since cancer has to be understood as a chronic disease in many cases: up to 70% of the patients live for at least 5 years after diagnosis [1, 2]; however, survival rates alone do not reflect the significant impairments patients suffer due to the disease and its treatment [3]. A substantial number of cancer survivors have to deal with physical and psychological burdens not only during active treatment, but also after hospital discharge when the long-term consequences of the disease and its treatment pose another challenge for their daily lives [4]. Common side effects of cancer treatment include emotional distress, pain and fatigue [4, 5] but cancer may also impact the patients’ occupational status [6, 7], or social functioning. Consequently, multidisciplinary cancer rehabilitation aiming at establishing the best possible physical, psychological and social conditions for cancer patients is increasingly recommended in national and international cancer treatment guidelines [8, 9]. Cancer rehabilitation in Austria is mainly offered at inpatient facilities with approximately 600 available beds [10]. Since the yearly incidence of about 39,000 new cancer cases is assumed to rise by 10% within the next 15 years [11] and cancer survival rates are steadily increasing, the need for cancer rehabilitation has most likely not yet reached its peak; therefore, rehabilitation programs need to be effective regarding treatment efficacy and costs. The EUROCHIP-3 Working Group on Cancer Rehabilitation has developed specific indicators to evaluate rehabilitation success, including quality of life (QOL), return to work, and satisfaction of specific rehabilitation needs (e. g. physical, psycho-oncological, dietary and speech and language therapy) [12]. Hence, to meet the requirements of successful cancer rehabilitation, the programs should consist of multidisciplinary efforts including medical, psychological and physiotherapeutic treatment as well as occupational therapy, dietetics, and social work [13].

There is a growing body of literature investigating the effects of inpatient cancer rehabilitation on health-related quality of life (HRQOL), psychological distress, and social functioning. Teichmann [14] reported statistically significant improvements with small effect sizes (ES) in physical and psychosocial HRQOL during inpatient rehabilitation, while no significant differences in the patients’ functional status were found. Patients who were more distressed at baseline benefited substantially more from rehabilitation programs. In a sample of breast cancer survivors, Hartmann et al. [15] compared the impact of two rehabilitation programs. In the standard care group, patients received a 3-week inpatient rehabilitation program with emphasis on psycho-oncological interventions. In the intervention group, patients received the same program with two additional 1‑week inpatient stays during the 12-month follow-up period. The HRQOL measured with the European Organisation for Research and Treatment of Cancer quality of life questionnaire (EORTC QLQ-C30) was defined as the study endpoint. Both groups showed clinically meaningful improvements with respect to global QOL, emotional and cognitive functioning, but no statistically significant differences were found between the two study arms. Another study including breast cancer survivors [16] reported a significant reduction of anxiety (ES = 0.50) and depression (ES = 0.37) during inpatient rehabilitation. In the study by Rath et al. [17] both patient samples in inpatient and outpatient rehabilitation reported significant improvements regarding the HRQOL, while no significant difference in the effectiveness between the settings was found. In a recent study changes in HRQOL in three cancer entities (breast, prostate and colon) during inpatient rehabilitation and a 3 months follow-up were analyzed [18]. Breast cancer and prostate cancer patients reported the largest improvements regarding emotional functioning during rehabilitation, while colon cancer patients showed the greatest increase in global QOL during rehabilitation. Regarding the symptom scales, breast and colon cancer patients reported the largest improvements for fatigue, while prostate cancer patients benefited the most regarding nausea and vomiting. Within the first 3 months after end of rehabilitation, emotional functioning decreased in all 3 samples, while physical and role functioning further increased for patients with prostate cancer and colon cancer.

Such studies from the literature suggest a substantial positive impact of multidisciplinary rehabilitation programs on HRQOL in cancer survivors; however, comparative evaluation of different rehabilitation programs did not result in the identification of a single superior type of rehabilitation. Furthermore, most of the studies were conducted in homogeneous cancer entities (e. g. breast cancer). The aim of our study was to investigate the change of psychological distress and HRQOL during a multidisciplinary inpatient rehabilitation. The rehabilitation program was characterized by a high level of treatment individualization and our study included a large mixed sample of cancer survivors, representing the real-world setting of cancer rehabilitation.

## Methods

### Sample and procedure

We retrospectively analyzed data collected in clinical routine from inpatients of the Oncological Rehabilitation Center, St. Veit im Pongau, Austria. Patients were routinely asked to complete a set of patient-reported outcome (PRO) measures at home prior to the beginning of the treatment (T0). The assessment instruments included sociodemographic data, information on previous treatment, treatment preferences as well as questionnaires on HRQOL (EORTC QLQ-C30) [19] and psychological distress using the hospital anxiety and depression scale (HADS) [20]. After the end of the rehabilitation treatment, patients were asked to complete the questionnaires again (T1). Inclusion criteria were: (a) any cancer diagnosis, (b) completed radiation and/or chemotherapy, (c) currently in full remission, (d) minimum age of 18 years and (e) completed assessments at both time points. Since some patients postponed the start of rehabilitation after completing the initial assessment (T0), patients were excluded from the analysis if the time interval between baseline assessment and admission to the rehabilitation center exceeded 4 weeks.

Except for an initial phase of 4 months using paper-pencil forms, data collection was done using the online software computer-based health evaluation system (CHES) [21]. This is a software for the administration of electronic patient-reported outcome (ePRO) assessments, storage of PRO, clinical and sociodemographic data, data import and export and can also be used for the graphical presentation of ePRO results in relation to reference values. To enable a gapless support after the inpatient rehabilitation, the pre-rehabilitation and post-rehabilitation HRQOL scores were included in the final report of the rehabilitation center, providing a table with selected HRQOL domains and a short explanatory comments, useful for interpretation by physicians in further treatment, such as general practitioners.

### Rehabilitation program

The rehabilitation center offers a multidisciplinary treatment schedule with different treatment options, which is adjusted to the patient’s needs according to the HRQOL and psychological distress data. The rehabilitation usually lasts 21 days and includes 2–3 h of treatment per day. The individualized and multidimensional therapy includes a combination of physical training, psycho-oncological interventions, lessons on general health behavior and on coping with cancer. Based on a specifically developed algorithm, which takes patients’ preferences for psychological treatment and their questionnaire scores into account, patients are assigned to different frequencies of psycho-oncological treatment at low (4 sessions/week), medium (5–6 sessions/week) or high (7–8 sessions/week) intensity. Patients can choose between different forms of psycho-oncological sessions, including sexual psychology or existential analysis. Furthermore, patients are offered various types of relaxation training, including progressive muscle relaxation, autogenic training and mindfulness training. Different types of occupational therapy are also available (single session or group).

Physical training includes different types of physiotherapy (e.g. manual therapy, the Bobath concept and pelvic floor exercise), massage (classical massage, lymph drainage and acupuncture), electrotherapy (electromyostimulation, ultrasound) as well as heat therapy and cryotherapy. Specific counseling (e. g. nutrition, smoking) is offered to improve health behavior during and after the rehabilitation treatment. Each patient has a case manager and a care manager for social counseling and for preparing a tailored care plan following the rehabilitation. A detailed list of the treatment elements used can be found in Table 2.

### Assessment instruments

The HRQOL was assessed using the EORTC QLQ-C30 [19], which is one of the most widely used cancer-specific HRQOL instruments in Europe. It consists of 30 questions building 5 functioning scales (physical, social, role, emotional, cognitive), 9 symptom scales (fatigue, nausea and vomiting, pain, dyspnea, sleep disturbances, appetite loss, constipation, diarrhea, and financial impact), and a scale for global QOL. Scoring was done according to the EORTC QLQ-C30 scoring manual. Raw scores were transformed to a scale from 0 to 100 with 100 reflecting the best possible score for functioning scales and the worst score for symptom scales.

Psychological distress was assessed using the HADS [20], which is a widely used screening instrument to detect psychological distress in patients with primary somatic disorders. It consists of 14 items which can be used to calculate a total score (range 0–42) as well as a depression (range 0–21) and an anxiety (range 0–21) subscale. Cut-off values can be used to differentiate non-cases (≤7), doubtful cases [8–10] and clinical cases (≥11) of anxiety or depressive disorder [22]. Good psychometric properties have been reported for the HADS [20, 22]. The German Cancer Society (Deutsche Krebsgesellschaft, DKG) recommends the HADS as a standard screening instrument for oncology patients. Patients with a HADS total score >15 were classified as distressed [23].

### Statistical analysis

Repeated measures analysis of variance (ANOVA) were used to investigate the change of psychological distress and HRQOL during the rehabilitation stay. Diagnosis, age and sex were included as covariates in the ANOVA. Furthermore, the time interval between assessment and start of treatment was entered as a covariate to control for potential interaction effects. The size of change before and after the rehabilitation was evaluated using Cohens’ *d*, and to evaluate the size of the interaction effects we calculated partial η^2^. Effect sizes (ES) were considered as small (d = 0.2, η^2^ = 0.01), medium (d = 0.5, η^2^ = 0.06) or large (d = 0.8, η^2^ = 0.14) [24, 25].

The mean values of both assessment points were compared to reference values for the EORTC QLQ-C30 [26] and the HADS [27] using independent sample t‑tests. For the scales of the EORTC QLQ-C30 a difference of 5–10 points, 10–20 points and >20 point indicate a small, moderate or large change, respectively [28]. A minimal important difference for the HADS anxiety and depression score of 1.3 and 1.4 points, respectively, has been reported by Puhan et al. [29]. Baseline sociodemographic and clinical characteristics of the drop-outs were compared to the included study sample using χ^2^-tests for nominal data and independent t‑tests for metric data. *P*-values <0.05 were considered statistically significant. For statistical analysis IBM SPSS (v22.0) was used.

## Results

### Patient characteristics

**Fig. 1:**
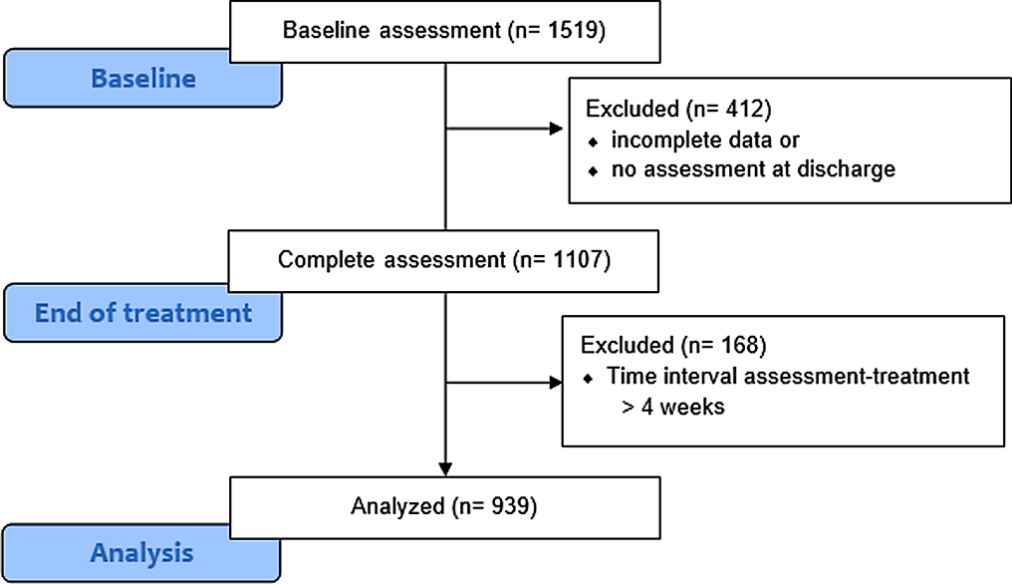
Flow chart showing inclusion and exclusion of patients in the study

The flow diagram in Fig. 1 shows the sample selection process. A total of 1519 patients who were treated during January 2014 and September 2015 completed the first assessment at home (paper-pencil or electronically) and 1107 (72.9%) completed both assessments. From those providing complete data, a further 168 patients were excluded because the time interval between the first assessment and therapy admission exceeded 4 weeks. The remaining 939 patients (61.8%) were included in the study.

The mean age of the remaining 939 patients was 58.6 years (SD 11.9) and 59.9% were women. The most frequent diagnoses were breast cancer (29.5%), hematological malignancies (10.5%), and colorectal cancer (10.0%). At baseline, 27.4% of the patients were psychologically distressed (HADS total score >15) and 21.9% had previous experience with psychotherapeutic treatment. Of the 49% of patients who wished for a psycho-oncological focus, 31% had no preferences regarding the specific type of psychological treatment. The mean time interval between first assessment and admission to the rehabilitation center was 8 days (SD 8 days). For further details see Table 1.

**Table 1:** Patient characteristics

Characteristics	Total sample *n* = 939 *n* (%)	
Mean age (years± SD)	58.6 (11.9), range:19–88	
*Sex*		
Women (%)	562	(59.9)
Men (%)	377	(40.1)
*Cancer sites*		
Breast cancer	277	(29.5)
Hemato-oncological malignancy	99	(10.5)
Colorectal cancer	94	(10.0)
Head and neck cancer	62	(6.6)
Prostate cancer (including penis and testes)	57	(6.1)
Gastric cancer (including esophagus)	52	(5.5)
Gynecological cancer	44	(4.7)
Lung cancer	43	(4.6)
Pancreatic cancer (including liver and gall bladder)	34	(3.6)
Urinary organs	34	(3.6)
Brain cancer	14	(1.5)
Others	68	(7.2)
Missing data	61	(6.5)
*HADS total score*		
Mean (SD)	11.9	7.2
*Psychotherapeutic pretreatment*		
Yes	204	(21.7)
No	726	(77.3)
Missing	9	(1.0)
*Preference for psycho-oncological focus*		
Yes	453	(48.2)
No	477	(50.8)
Missing	9	(1.0)
*Preferred psycho-oncological focus*		
Sexual psychology	49	(10.8)
Existential analysis	130	(28.7)
Biofeedback	99	(21.9)
No preference	127	(28.0)
Missing	48	(10.6)

Patients who had incomplete data or did not take part in the second assessment did not statistically differ from the included sample regarding sex (*p* = 0.22), diagnosis (*p* = 0.23) or level of psychological distress (*p* = 0.48). Non-participants were significantly older than the study sample (59.7 years vs. 58.1 years; *p* = 0.03). The mean duration of inpatient stay was 27 days (SD 9 days). Almost all patients received physical therapy and/or psycho-oncological treatment, while others were offered on an individual basis (e.g. health psychological group counseling and social counseling). A detailed treatment overview can be found in Table 2.

**Table 2:** Applied treatment interventions

Treatment			Treatment frequency per patient	
	*n*	(%)	*Median*	*IQR*
Physiotherapy/physical therapy^a^	905	(96.4)	34	23–30
Health psychological counseling (group, 60 min.)	904	(96.3)	1	1–1
Nutrition counseling	901	(96.0)	2	1–3
Psycho-oncology (single, 25 min.)	877	(93.4)	5	4–6
Massage/lymph drainage	855	(91.1)	5	4–6
Thermotherapy	791	(84.2)	6	4–7
Hydrogymnastics	445	(47.4)	4	3–5
Self-help training	412	(43.9)	3	2–3
Creativity training	403	(42.9)	5	3–6
Electrotherapy	383	(40.8)	6	4–8
Social counseling	330	(35.1)	1	1–1
Biofeedback/relaxation exercise	141	(15.0)	3	3–4
Ultrasonic therapy	37	(3.9)	4	3–6

*n* number of patients that received specific treatment at least once

^a^clustered category, including physiotherapy, gymnastics, back exercises, weight training

*IQR* interquartile range

## Improvement of HRQOL and psychological distress after rehabilitation

Comparison of patients’ PRO scores before and after rehabilitation showed clinically meaningful improvements in all domains of HRQOL, except dyspnea, with a ES up to 0.78. The largest improvements were found for emotional functioning (20.1 points), fatigue (17.2 points), and social functioning (17.1 points). Patients also reported a statistically significant (*p* < 0.001) and clinically meaningful decrease of anxiety (2.0 points) and depression (2.3 points) after treatment with ESs of d = 0.51 and d = 0.58, respectively. The percentage of patients above the clinical cut-off for anxiety dropped from 33.2% to 15.5%, and from 30.7% to 10.4% for depression. Details can be found in Tables 3 and 4.

**Table 3:** QLQ-C30 and HADS mean values at T0 and T1, effect sizes and reference values (general population)

	Study sample		Reference value			
	T0	T1		Mean difference		
	Mean (SD)	Mean (SD)	Mean (SD)	(T1-T0)	ES	*p* value
*HADS*						
Anxiety	6.1 (3.9)*	4.2 (3.3)*	4.7 (3.5)	**1.9**	0.49	<0.001
Depression	5.6 (4.0)*	3.4 (3.2)*	4.8 (4.0)	**2.2**	0.56	<0.001
*QLQ-C30 functioning*						
Physical functioning	72.3 (21.0)*	79.8 (19.6)*	92.2 (15.1)	**7.5**	0.36	<0.001
Role functioning	58.0 (31.0)*	73.0 (26.4)*	90.4 (20.2)	**15.0**	0.48	<0.001
Social functioning	58.8 (30.7)*	75.9 (25.8)*	93.4 (17.2)	**17.1**	0.56	<0.001
Emotional functioning	58.7 (25.6)*	78.7 (21.3)*	83.5 (19.7)	**20.1**	0.78	<0.001
Cognitive functioning	72.8 (27.3)*	78.6 (23.8)*	93.5 (14.5)	**5.8**	0.21	<0.001
*Global quality of life*	58.6 (20.3)*	74.2 (17.6)*	75.0 (19.6)	**15.6**	0.76	<0.001
*QLQ-C30 symptoms*						
Fatigue	53.0 (26.5)*	35.8 (23.5)*	15.5 (21.6)	**17.2**	0.65	<0.001
Nausea/vomiting	13.4 (22.4)*	7.1 (17.2)*	2.2 (8.9)	**6.3**	0.28	0.17
Pain	40.2 (29.3)*	27.5 (26.4)*	16.7 (24.2)	**12.7**	0.43	<0.001
Dyspnea	33.8 (32.7)*	29.3 (27.5)*	7.5 (19.3)	4.5	0.13	0.04
Insomnia	46.3 (33.9)*	36.7 (32.3)*	12.4 (23.3)	**9.6**	0.28	<0.001
Appetite loss	26.2 (33.2)*	14.2 (26.2)*	3.8 (13.3)	**12.0**	0.36	<0.001
Constipation	20.7 (31.3)*	11.5 (24.2)*	2.2 (10.3)	**9.2**	0.29	<0.001
Diarrhea	17.2 (27.7)*	11.5 (22.8)*	2.5 (11.6)	**5.7**	0.21	<0.001
Financial difficulties	30.0 (34.0)*	20.7 (29.8)*	4.8 (16.6)	**9.3**	0.27	0.001

Difference = absolute value; clinical meaningful changes [24, 25] in bold type

*ES *effect size

*Significant difference (*p* < 0.05) to reference values

**Table 4:** HADS anxiety (HADS-A) and depression (HADS-D) severity pretreatment and posttreatment with χ^2^-values

	T0		T1		
	*N*	(%)	*N*	%	*p*-value
*HADS-A*					
Non-cases (≤7)	624	(66.8)	790	(84.1)	<0.001
Doubtful cases (8–10)	178	(19.1)	108	(11.5)	
Clinical cases (≥11)	132	(14.1)	38	(4.0)	
*HADS-D*					
Non-cases (≤7)	648	(69.3)	837	(89.5)	<0.001
Doubtful cases (8–10)	158	(16.9)	63	(6.7)	
Clinical cases (≥11)	129	(13.8)	35	(3.7)	

To test if the specific cancer entity, sex, age or the time interval between assessment and treatment influenced the detected improvements, interaction effects were considered in the calculations. We did not find statistically significant associations of patient characteristics (age, sex, diagnosis) with the QLQ-C30 and HADS scales, with only two exceptions: older patients showed stronger improvement on the QLQ-C30 physical functioning domain (*p* = 0.04) and less improvement for HADS anxiety (*p* = 0.01). We also investigated the impact of time elapsed between baseline assessment and admission to evaluate the impact of natural recovery as opposed to recovery during the rehabilitation process. We only found small ES for such an impact (all partial η <0.02), i. e. patients who completed the baseline assessment prior to admission (up to 4 weeks) did not show substantially different change rates in the study period compared to patients who completed the assessment at admission.

## Comparison of pre-rehabilitation and post-rehabilitation HRQOL and psychological distress scores to GP

At baseline, the sample reported clinically meaningful large differences (>20 points) in their HRQOL compared to the general population (GP) in 10 out of the 15 QLQ-C30 domains (*p* < 0.001). The largest differences were found regarding fatigue (37.5 points), social functioning (34.6 points), and insomnia (33.9 points) and the smallest differences were found for nausea (11.2 points) and diarrhea (14.7 points). Compared to the GP, differences in the mean scores were clinically meaningful for anxiety (1.4 points) but not for depression (0.8 points). The largest proportion of psychologically distressed patients at baseline were found in patients with secondary or ill-defined tumors (41.4%), lung cancer (37.2%), brain cancer (35.7%) and the lowest proportion was found in patients with colorectal cancer (13.8%).

**Fig. 2:**
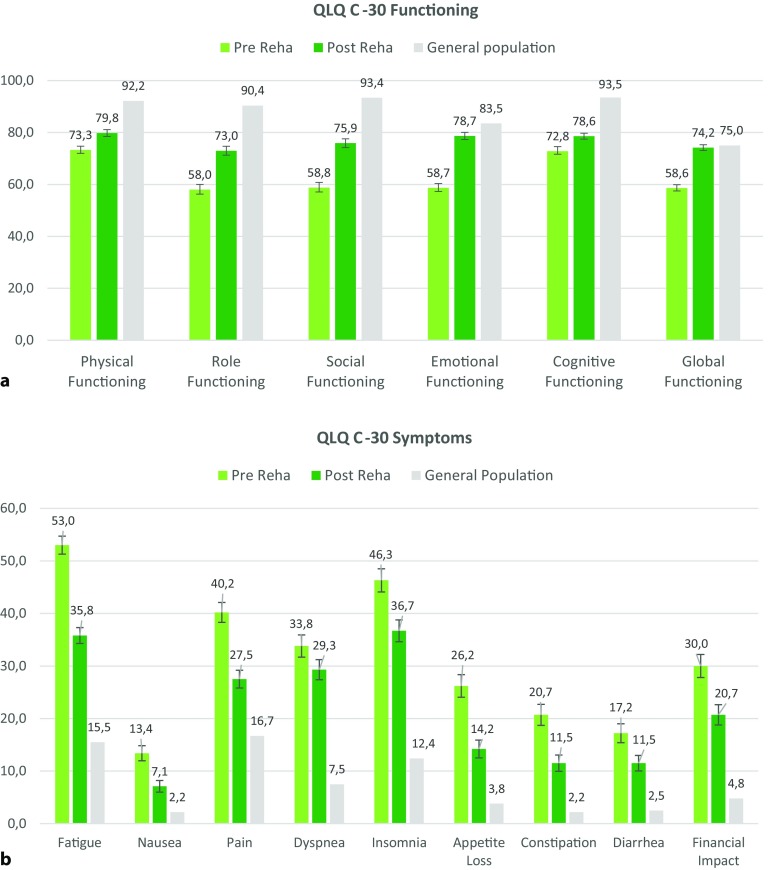
Mean QLQ-C30 values before and after rehabilitation (with 95% confidence intervals) and mean values for the general population. **a** QLQ-C30 functioning scales, **b** QLQ-C30 symptom scales

At the end of treatment, no clinically meaningful differences regarding emotional functioning (4.8 points), nausea (1.0 point), and global quality of life (4.9 points) and only small differences in constipation (9.3 points) and diarrhea (9.0 points) were found compared to the GP. Although patients overall reported improvement in the HRQOL, they still had significantly lower values especially regarding insomnia (24.3 points), dyspnea (21.9 points), and fatigue (20.3) than the GP. While the mean anxiety scores after treatment were comparable to the GP, the mean depression scores were even lower than in the GP, with a clinically meaningful difference of 1.4 points (d = 0.4). Fig. 2 shows the patients’ HRQOL status before and after treatment compared to the GP.

## Discussion

In the present study, we investigated HRQOL, anxiety and depression scores before and after a multidisciplinary inpatient cancer rehabilitation program lasting 3–4 weeks in a broad mixed sample of cancer survivors. Rehabilitation included various aspects of physical and psychological treatment, but also dietary counseling, social work, and occupational therapy. Patient scores significantly improved in all domains of HRQOL as assessed by the EORTC QLQ-C30 in the course of the rehabilitation. The largest differences were found for emotional functioning (20 points), social functioning (18 points), fatigue (18 points) and pain (13 points). Moreover, the anxiety and depression scores significantly decreased. The type of cancer, age, and sex had no influence on the improvements found for the QLQ C‑30 and HADS mean scores.

### Improvement of functional status

The largest improvement was found for emotional functioning with an increase of 20 points. This is in accordance with previous studies, which also found large improvements of emotional functioning during rehabilitation [18, 30]. Many cancer survivors reported impaired emotional functioning as a result of the cancer disease and its treatment [4, 5], which can be effectively treated by psycho-oncologists [31]. Patients also reported very large improvements in social functioning. Concerning the sustainability of such improvements, results are ambiguous. Previous studies reported a further decline of the initial improvement of social function within the first 3 months after rehabilitation in patients with breast cancer [18, 30], while others found that the improvement remained stable in patients with prostate cancer, and even improved in patients with colon cancer within the first 3 months after rehabilitation [18]. So far, only very limited knowledge regarding the long-term effectiveness of inpatient rehabilitation on social function is available. We found comparably smaller but nevertheless clinically meaningful changes regarding physical functioning and cognitive functioning, which are in line with previous studies [15, 18, 30]. Compared to other aspects of HRQOL, patients already reported relatively high functioning at baseline, leaving less room for improvement. Previous studies showed that improvements achieved during the rehabilitation tend to further increase in the follow-up period [15, 18]. Nevertheless, the implementation of specific interventions targeted at cognitive functioning (e. g. memory and concentration) may lead to larger changes in this area. Especially in respect to physical functioning and cognitive functioning, web-based self-management and self-help interventions [32, 33] may be feasible and affordable ways to stabilize and further improve positive effects achieved during inpatient rehabilitation.

Lamprecht et al. [18] suggested to take the socioeconomic situation of the patients into account in rehabilitation treatment, because the risk of unemployment is particularly high in cancer patients [34]. This can have strong negative influence on patients QOL. In our study, we found a significant decrease of perceived financial difficulties before and after rehabilitation.

Our patients reported relatively large improvements in their global QOL. Global QOL assessments are often seen as an appropriate alternative to a broader, multidimensional approach since the time burden may result in poor completion rates [35]. Nevertheless, it has been repeatedly pointed out that global measures of QOL have less clinical value for assessing change than broader measures, since the source of the change in a global score cannot clearly be determined and it is not comprehensible which aspects of QOL patients refer to when they were asked to rate their overall QOL [36]. The HRQOL scores of our sample strongly differed from the general population at the end of rehabilitation in all specific functioning subscales but not global QOL. This shows that although the assessment of global QOL may roughly indicate an overall change in patients’ health status, broad and differentiated assessment of QOL is advisable, since global QOL scales run the risk of misjudging patients’ functioning and symptoms.

### Improvement of symptom scores

At baseline, our patient sample reported clinically meaningful worse symptom scores, not only compared to the general population [26], but also compared to previous studies about inpatient cancer rehabilitation [18]. After rehabilitation, we found clinically significant improvement in almost all symptom domains assessed by the QLQ-C30, with the largest differences in fatigue, pain, and appetite loss. Although patients reported large improvements in all symptom domains assessed by the QLQ-C30, at the end of their rehabilitation stay their scores were still significantly worse than those of the general population. This shows that there is still a need to evaluate how cancer survivors can be further supported after the end of their inpatient rehabilitation stay. A first step would be to investigate the development of patients’ health status within the first year after rehabilitation to detect whether certain domains of HRQOL need to be specifically targeted.

### Improvement of psychological distress

Cancer survivors in inpatient rehabilitation have a high prevalence of mood disorders [37] and use psychological care significantly more often than cancer patients in acute care hospitals [38]. Consistent with previous research, we found significantly higher anxiety and depression scores than the general population at baseline [27] but neither anxiety nor depression significantly differed across cancer entities. After rehabilitation, patients reported highly significant and clinically meaningful reduction of both anxiety and depression scores. The effect size of decreased anxiety was comparable to previous studies, while the improvement of depression was even larger [16, 39]. At baseline 14.1% of the sample showed clinically relevant anxiety and 13.8% clinically relevant depression. The percentage dropped in both cases to 4% after treatment. After the rehabilitation, the mean depression and anxiety values of our sample were even lower than the mean values of the general population.

### Strengths and limitations

Our study has several strengths. We included a broad, unselected sample of cancer survivors in a multidisciplinary treatment setting as opposed to previous studies which often included highly selected trial samples and evaluated only specific interventions. The data used in the study were collected in the course of routine treatment and we presented detailed description of the interventions which patients engaged in during rehabilitation. Consequently, our study represents a real-world cancer rehabilitation setting.

The present study also has some limitations, which restrict the generalization of the findings. Firstly, the study is limited by its observational nature and the fact that no control group was used in the study. This is because our calculations are based on a retrospective analysis of data obtained from routine clinical assessment in an inpatient cancer rehabilitation center, where typically no control groups are available. Since we included a relatively large number of unselected patients seeking routine treatment in our analyses, we consider the sample to be representative of patients in routine inpatient rehabilitation centers. Due to the missing control group we cannot rule out that the difference in the PRO scores before and after treatment are partially influenced by natural changes over time. Nevertheless, the observed and partly large improvements during the rehabilitation period warrant further investigation in a controlled design, allowing conclusions to be drawn on the effectiveness of treatment. Secondly, the varying time interval between the baseline assessment and the start of the rehabilitation treatment might interact with the treatment outcome. We therefore excluded all patients from the analysis, who started the rehabilitation stay more than 28 days after their baseline assessment. Furthermore, we included the time interval as a covariate in the ANOVA to investigate interaction effects. We did not, however, find any significant influence of the time interval on the improvement of pre-post QLQ C‑30 and HADS scores after excluding patients with more than 28 days between assessment and admission. Thirdly, non-participants were slightly older than the study sample but the difference was of negligible size. We also found interaction effects for age, but because of the very low ES the influences on the main results were also considered negligible. Finally, in the present study no follow-up assessment was included, which limits our ability to draw conclusions with respect to the long-term development of the patients HRQOL and psychological distress after rehabilitation treatment. This is a common problem in the evaluation of cancer rehabilitation and strongly restrains the evidence base for the long-term effects of cancer rehabilitation [40]; however, it has to be mentioned that we are currently conducting a follow-up study, aiming to assess HRQOL of patients at 3, 6 and 12 months after the rehabilitation treatment.

## Conclusion

In conclusion, our results showed large improvements of patients’ HRQOL and psychological distress after a multidisciplinary oncological inpatient rehabilitation. The observed improvements warrant further prospective and controlled studies to evaluate the sustainability of the treatment effect and the long-term effectiveness of inpatient rehabilitation.
